# Ethylenediamine grafted to graphene oxide@Fe_3_O_4_ for chromium(VI) decontamination: Performance, modelling, and fractional factorial design

**DOI:** 10.1371/journal.pone.0187166

**Published:** 2017-10-30

**Authors:** Xinjiang Hu, Jiawen Xu, Cuiyu Wu, Jianbin Deng, Wenwei Liao, Yuxiang Ling, Yuanxiu Yang, Yina Zhao, Yunlin Zhao, Xi Hu, Hui Wang, Yunguo Liu

**Affiliations:** 1 College of Environmental Science and Engineering, Central South University of Forestry and Technology, Changsha, P.R. China; 2 Faculty of Life Science and Technology, Central South University of Forestry and Technology, Changsha, P.R. China; 3 College of Natural Resources and Environment, South China Agricultural University, Guangzhou, P.R. China; 4 Institute of Bast Fiber Crops, Chinese Academy of Agricultural Sciences, Changsha, P.R. China; 5 College of Environmental Science and Engineering, Hunan University, Changsha, P.R. China; 6 Key Laboratory of Environmental Biology and Pollution Control (Hunan University), Ministry of Education, Changsha, P.R. China; Institute of Materials Science, GERMANY

## Abstract

A method for grafting ethylenediamine to a magnetic graphene oxide composite (EDA-GO@Fe_3_O_4_) was developed for Cr(VI) decontamination. The physicochemical properties of EDA-GO@Fe_3_O_4_ were characterized using HRTEM, EDS, FT-IR, TG-DSC, and XPS. The effects of pH, sorbent dose, foreign anions, time, Cr(VI) concentration, and temperature on decontamination process were studied. The solution pH can largely affect the decontamination process. The pseudo-second-order model is suitable for being applied to fit the adsorption processes of Cr(VI) with GO@Fe_3_O_4_ and EDA-GO@Fe_3_O_4_. The intra-particle diffusion is not the rate-controlling step. Isotherm experimental data can be described using the Freundlich model. The effects of multiple factors on the Cr(VI) decontamination was investigated by a 2^5−1^ fractional factorial design (FFD). The adsorption process can significantly be affected by the main effects of A (pH), B (Cr(VI) concentration), and E (Adsorbent dose). The combined factors of AB (pH × Cr(VI) concentration), AE (pH × Adsorbent dose), and BC (Cr(VI) concentration × Temperature) had larger effects than other factors on Cr(VI) removal. These results indicated that EDA-GO@Fe_3_O_4_ is a potential and suitable candidate for treatment of heavy metal wastewater.

## Introduction

Heavy metal pollution is a current worldwide environmental concern because it can harm ecosystems and endanger human health. Since the industrial revolution, chromium has been widely used in electroplating, tanning, dying, smelting, and corrosion protection [1–3]. Cr(VI), one form of chromium, is very harmful to most organisms due to its mammalian toxicity and carcinogenicity [4]. Therefore, it is necessary and important to separate Cr(VI) ions from aqueous solution before they are discharged into aquatic systems.

Compared with traditional chemical precipitation and ion exchange methods, adsorption is a simpler, faster, and more economically viable method for removing heavy metals from various wastewaters [5–7]. The nature of an adsorbent is critical to the adsorption process, and the efficiency and cost of which are determined by the efficiency of the adsorbent regarding contaminant removal and its solid-liquid separation ability [1]. Therefore, it is desirable to find adsorbents that possess both high adsorption ability and straightforward solid-liquid separation.

In recent years, graphene oxide (GO) has been used as an excellent adsorbent material due to its unique properties [8, 9]. GO has very high surface area and a large number of carboxyl, hydroxyl, carbonyl, and epoxy groups [8, 10], which can be used as anchoring sites for metal ions. GO and GO-based materials have been used as adsorbents for binding metals such as chromium [11], cadmium [12], lead [13], zinc [14], platinum [15], and copper [16]. However, due to its nanoscale and hydrophilic nature, GO is difficult to separate from aqueous solution following the adsorption process. The dispersion of magnetic nanomaterials on GO sheets is a topic of current research because it combines the advantages of high adsorption rate and easy phase separation [17, 18]. Therefore, it is important to integrate graphene oxide with magnetic nanomaterials for improving the solid-liquid separation capacity of the composite[19]. The adsorption capacity of an adsorbent for contaminants is partly determined by the number of functional groups [20]. Ethylenediamine is low-toxicity and low-cost, and contains two amino groups that can form stable chelates with metal ions. Therefore, grafting ethylenediamine to GO and GO-based materials may increase their adsorption ability. However, the adsorption behaviors of ethylenediamine modified magnetic graphene oxide composite (EDA-GO@Fe_3_O_4_) for Cr(VI) ions have not been fully investigated.

It is well known that environmental factors including pH, contact time, temperature, initial metal concentration, and background electrolyte species may affect the efficiency of an adsorbent for metal ions, and that this could be increased by optimizing these factors [21]. Traditional one-factor experimental design just study one factor at a time, which cannot investigate the interaction of factors [22]. Full factorial experimental design can give information about the interaction of factors, but it is only suitable for experiments with a small number of factors [21]. Fractional factorial design (FFD) can identify significant factors and assess interaction of factors only with a smaller number of experiments [21, 23]. Besides, the produced results can be easily analyzed without any complicated calculations. Therefore, it is significant to identify the key factors that have large effects on the Cr(VI) decontamination by EDA-GO@Fe_3_O_4_ using FFD.

In this study, a novel type of GO based composite named EDA-GO@Fe_3_O_4_ was developed for effective Cr(VI) decontamination. To the authors’ knowledge, few studies attempted to graft ethylenediamine to magnetic graphene oxide for improving the Cr(VI) removal efficiency. Moreover, there has not been any studies on the use of FFD to identify the main factors influencing adsorption efficiency of EDA-GO@Fe_3_O_4_ for Cr(VI) ions. The aims of this research are to: (1) synthesize and characterize magnetic hybrid adsorbent (EDA-GO@Fe_3_O_4_) and apply it for removing Cr(VI) ions from wastewater; (2) evaluate the effects of pH, sorbent dose, foreign anions, time, Cr(VI) concentration, and temperature on removal process; (3) investigate the reusability of EDA-GO@Fe_3_O_4_ composite; (4) apply kinetics and isotherm models for modelling the adsorption experiments; and (5) use FFD to identify significant factors and interactions for removing Cr(VI) ions with EDA-GO@Fe_3_O_4_.

## Materials and methods

### Materials

The chemicals such as H_2_SO_4_, P_2_O_5_, NaNO_3_, FeCl_2_·4H_2_O, FeCl_3_·6H_2_O, NH_3_·H_2_O, H_2_O_2_ were supplied by Guangzhou Chemical Reagent Factory. K_2_S_2_O_8_ was purchased from Tianjin Damao Chemical Reagent Factory. Graphite powder, ethylenediamine, K_2_Cr_2_O_7_, and KMnO_4_ were obtained from Tianjin Fuchen Chemical Reagent Factory. All reagents above were of analytical grade.

### Synthesis of EDA-GO@Fe_3_O_4_

The GO was prepared using the modified Hummers procedure reported previously [17]. Natural graphite was first preoxidized with K_2_S_2_O_8_, P_2_O_5_, and H_2_SO_4_, then further oxidized with H_2_SO_4_, NaNO_3_, and KMnO_4_. Lastly, the graphite oxide layers were separated by ultrasonication to obtain a GO suspension.

Coprecipitation method was used to synthesize the magnetic graphene oxide (GO@Fe_3_O_4_). Fe^2+^and Fe^3+^ were added to the GO suspension and stirred vigorously for 2 min. Next, concentrated NaOH solution (100 g/L) was added into the mixture until the solution pH was 10, then the mixture was stirred constantly for 45 min at 85°C. The product was rinsed with Milli-Q water to obtain a black-colored GO@Fe_3_O_4_ suspension.

Grafting ethylenediamine to the magnetic graphene oxide composite (EDA-GO@Fe_3_O_4_) was achieved by modifying GO@Fe_3_O_4_ with ethylenediamine [24]. First, 9.0 mL ammonia solution was added to the GO@Fe_3_O_4_ suspension and stirred for 5 min. Then 36 mL ethylenediamine was added into the suspension and stirred for 10 min. Next, the suspension was stirred at 95°C for 6 h. Finally, ethanol and Milli-Q water were used to wash the product to neutral pH.

### Characteristics of EDA-GO@Fe_3_O_4_

High-resolution transmission electron microscopy (HRTEM) of the EDA-GO@Fe_3_O_4_ was collected with a Tecnai G2-F20 (FEI, USA). The EDS spectrum was collected with an energy-dispersive X-ray spectrometer (FEI, USA). The FT-IR spectrum of EDA-GO@Fe_3_O_4_ was collected using a Magna-IR 170 spectrometer with KBr pellets at room temperature (Nicolet, USA). TG and DSC curves were recorded using a Q600 thermoanalyzer (TA, USA). The surface elemental composition was analyzed using an ESCALAB 250Xi X-ray photoelectron spectroscope with a resolution of 0.5 eV (Thermo, USA).

### Adsorption experiments

#### Batch adsorption experiments

Adsorption experiments were study in a water bath shaker. The EDA-GO@Fe_3_O_4_ or GO@Fe_3_O_4_ and the Cr(VI) solution were added to 100 mL Erlenmeyer flasks. 0.01 or 0.1 M NaOH and HCl solution was used to adjust the pH values of the suspensions. Then, the Erlenmeyer flasks were shaken for 8 h at the desired temperature. After the adsorption process, a permanent magnet was used to separate the suspension. The concentration of Cr(VI) ions was determined by a UV spectrophotometer at 540 nm [20]. The adsorption capacities (*q*_e_, mg/g) and adsorption percentages (*E*_e_, %) of EDA-GO@Fe_3_O_4_ or GO@Fe_3_O_4_ were calculated by the following equations:

$$q_{e}=\frac{(C_{0}—C_{e})V}{W}$$ (1)

$$E_{e}=\frac{(C_{0}—C_{e})\times 100}{C_{0}}$$ (2)

where *C*_0_ (mg/L) is the initial Cr(VI) concentration; *C*_e_ (mg/L) is the equilibrium concentration of Cr(VI); *V* (L) is the volume of the Cr(VI) solution; and *W* (g) is the dosage of EDA-GO@Fe_3_O_4_ or GO@Fe_3_O_4._

#### Two-level fractional factorial design

Five factors (A: pH, B: Cr(VI) concentration, C: temperature, D: time, E: adsorbent dose) were screened for their effects on *q*_e_ (response) by a 2^5−1^ FFD with resolution V. The experimental design matrix and corresponding values of each factor are shown in S1 Table. Design Expert 8.0.6 (Stat-Ease Inc., USA) and Minitab Release 16 (Minitab Inc., USA) were used for the FFD of the experiments and regression analysis of the experimental data obtained.

### Modeling of adsorption kinetics and isotherm

#### Adsorption kinetics

The pseudo-first-order, pseudo-second-order, and intra-particle diffusion models can be expressed with Eqs 3, 4 and 5, respectively [25, 26].

$$q_{t}=q_{e}(1-e^{-k_{1}t})$$ (3)

$$q_{t}=\frac{q_{e}^{2}k_{2}t}{1+q_{e}k_{2}t}$$ (4)

$$q_{t}=k_{p}t^{1/2}+C$$ (5)

where *q*_t_ (mg/g) is the adsorption capacity of EDA-GO@Fe_3_O_4_ or GO@Fe_3_O_4_ at time *t* (h); *k*_1_ (1/min), *k*_2_ (g/mg min), and *k*_p_ (mg/g min^0.5^) are the adsorption rate constants for the three kinetic models, respectively; *q*_e_ (mg/g) is the adsorption capacities calculated by the kinetics models; *C* of adsorption constant is the intercept.

#### Adsorption isotherm

The nonlinear form of Langmuir, Freundlich, Temkin isotherm models are given by the Eqs 6, 7 and 8, respectively [27–29].

$$q_{e}=\frac{q_{\max}K_{L}C_{e}}{1+K_{L}C_{e}}$$ (6)

$$q_{e}=K_{F}C_{e}^{1/n}$$ (7)

$$q_{e}=\frac{RT}{b_{T}}\ln(a_{T}C_{e})$$ (8)

where *q*_e_ (mg/g) is the adsorption amount of Cr(VI) ions; *q*_max_ (mg/g) is the maximum adsorption capacities of the adsorbent; *K*_L_ (L/mg), *K*_F_ and *n*, *a*_*T*_ (L/g) and *b*_*T*_ (kJ/mol) are the constants for the Langmuir, Freundlich, and Temkin isotherm models, respectively; *C*_e_ (mg/L) is the equilibrium concentration after the adsorption process; *T* (K) is the temperature; and *R* (8.314 × 10^−3^ kJ/mol K) is the gas constant.

## Results and discussion

### Characterization

**Fig 1:**
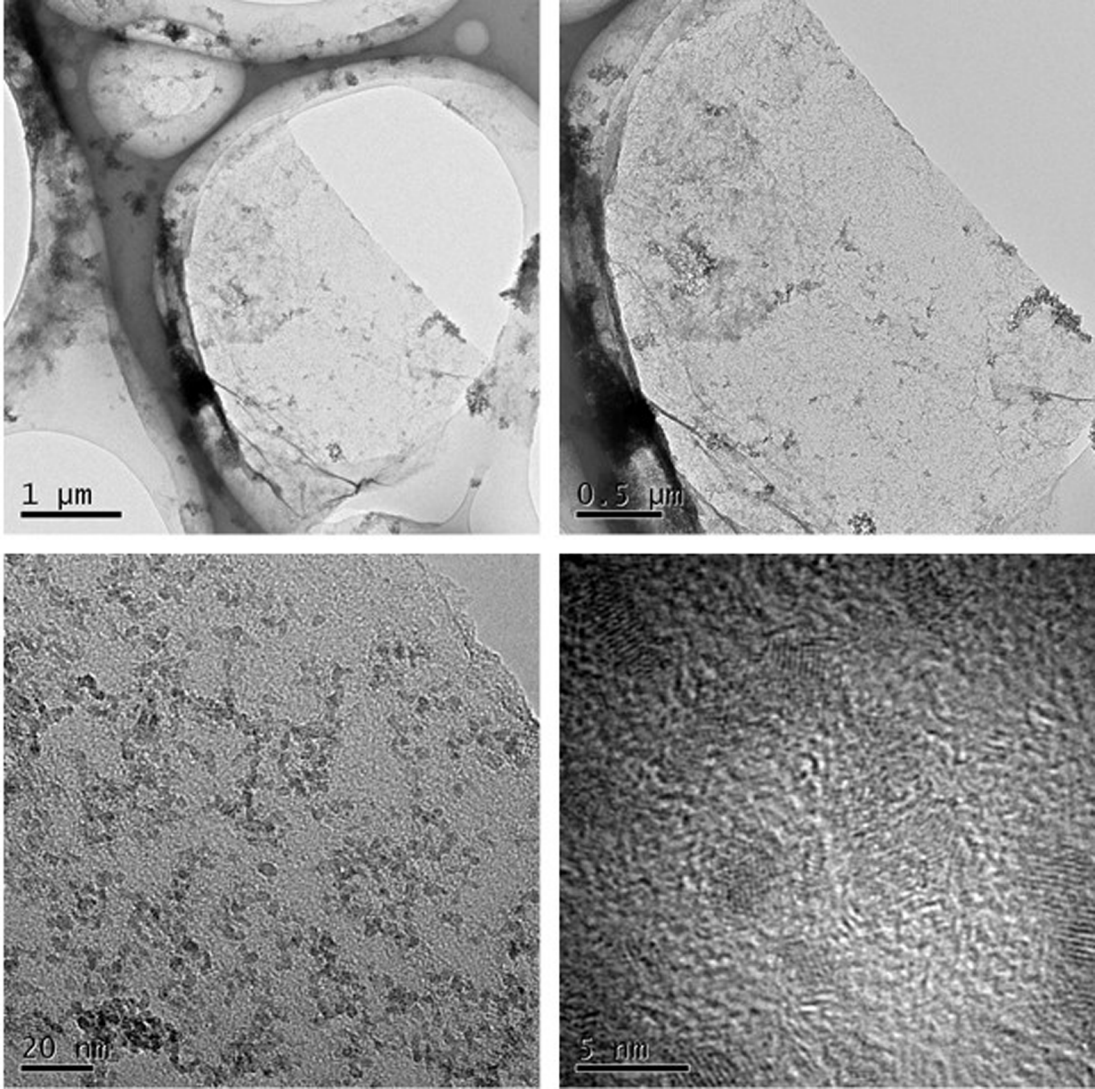
HRTEM images of EDA-GO@Fe_3_O_4_ at different magnification.

The morphology and microstructure of EDA-GO@Fe_3_O_4_ were characterized by HRTEM, and images at different magnifications are shown in Fig 1. EDA-GO@Fe_3_O_4_ revealed a typical fabric-like shape with a two-dimensional nanosheet structure (Fig 1A and 1B). From Fig 1, several small black spots (Fe_3_O_4_ nanoparticles) are dispersed on the GO nanosheets.

**Fig 2:**
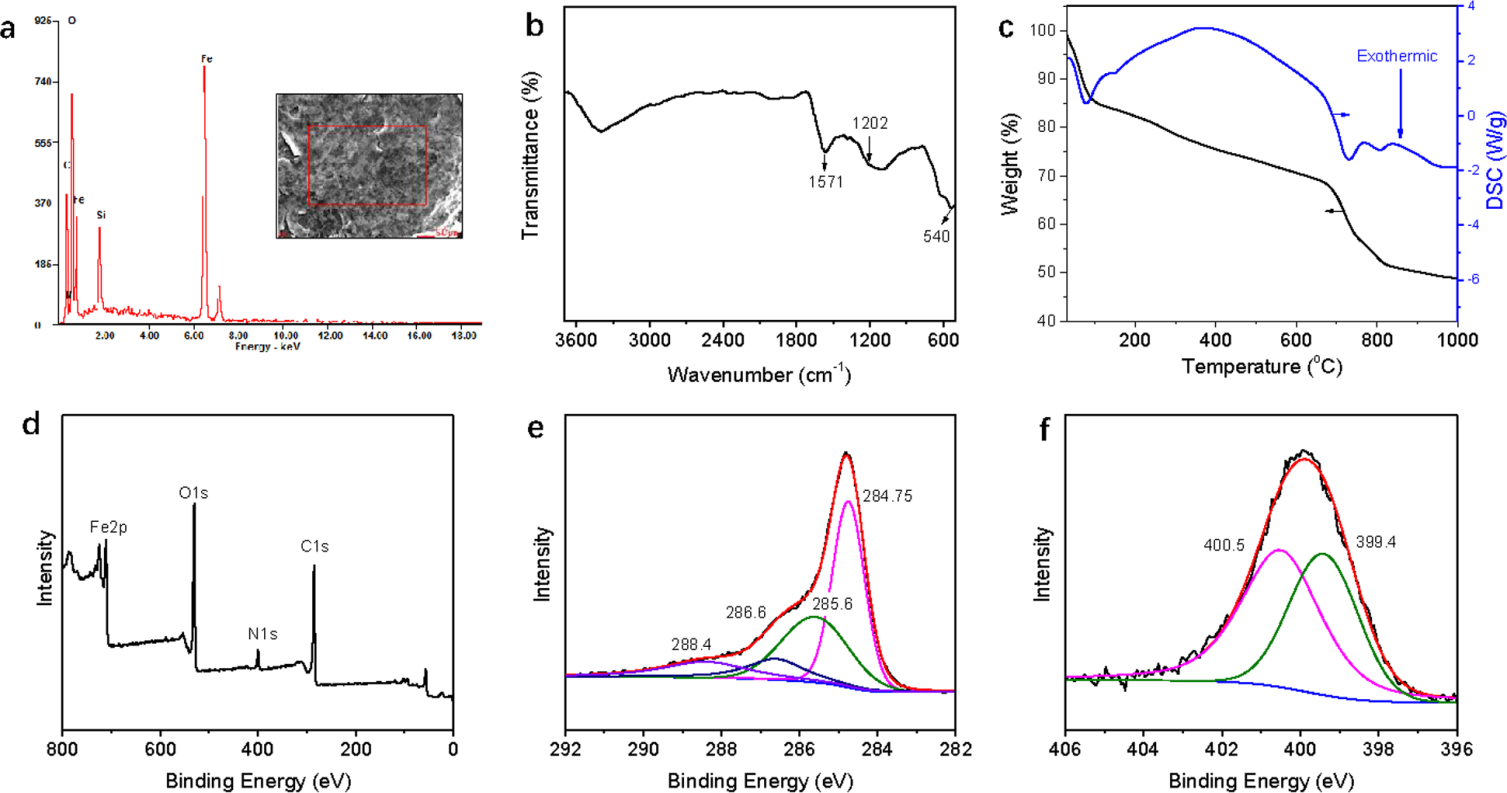
(a) EDS, (b) FT-IR, (c) TG-DSC, (d) XPS survey scan spectrum, (e) C1s and (f) N1s XPS spectra of EDA-GO@Fe_3_O_4_.

The EDS spectrum (Fig 2A) indicated that Fe, C, O, and N were present. Carbon came mainly from the GO nanosheets and the oxygen from the oxygen-containing functional groups in GO and Fe_3_O_4_ nanoparticles. N arose mainly from the amino groups in the grafted ethylenediamine, which indicated that ethylenediamine had been successfully introduced into the GO@Fe_3_O_4._

Fig 2B shows the FT-IR spectrum of EDA-GO@Fe_3_O_4_ composite. The peak at 540 cm^-1^ is attributed to Fe–O in Fe_3_O_4_, indicating successful connections between the Fe_3_O_4_ nanoparticles and the GO nanosheets. The peaks at 1571 cm^-1^ and 1202 cm^-1^ are attributable to the N–H stretching vibration (in the C–NH group) and C–N (in the–C–NH–C–and–NHCO–groups), respectively [24].

The TG-DSC curves of EDA-GO@Fe_3_O_4_ are shown in Fig 2C. The 15% loss of mass between 30°C and 106°C was attributed to the evaporation of water. The 51% loss of mass observed when the temperature ranged from 106°C to 1000°C was ascribed to pyrolysis of the grafted ethylenediamine and the oxygen-containing functional groups on the surfaces of the EDA-GO@Fe_3_O_4_ composite.

In order to gain further information on the chemical composition of EDA-GO@Fe_3_O_4_, XPS analysis was performed; the results are shown in Fig 2D–2F. From the XPS survey scan spectrum of EDA-GO@Fe_3_O_4_ (Fig 2D), four distinct peaks corresponding to C1s, O1s, Fe2p, and N1s were obtained. The N1s originated largely from the grafted ethylenediamine. The C1s spectrum of EDA-GO@Fe_3_O_4_ (Fig 2E) was separated into four different peaks at binding energies of 284.75, 285.6, 286.6, and 288.4 eV, attributing to C–C, C–N, C–O and C = O groups, respectively [24]. Fig 2F shows that the N1s spectrum of EDA-GO@Fe_3_O_4_ separated into two peaks centered at 399.4 eV and 400.5 eV, corresponding to the N in the amine and amide, respectively [30]. These results indicated that the surfaces of GO@Fe_3_O_4_ were successfully modified by ethylenediamine.

### Effect of pH

**Fig 3:**
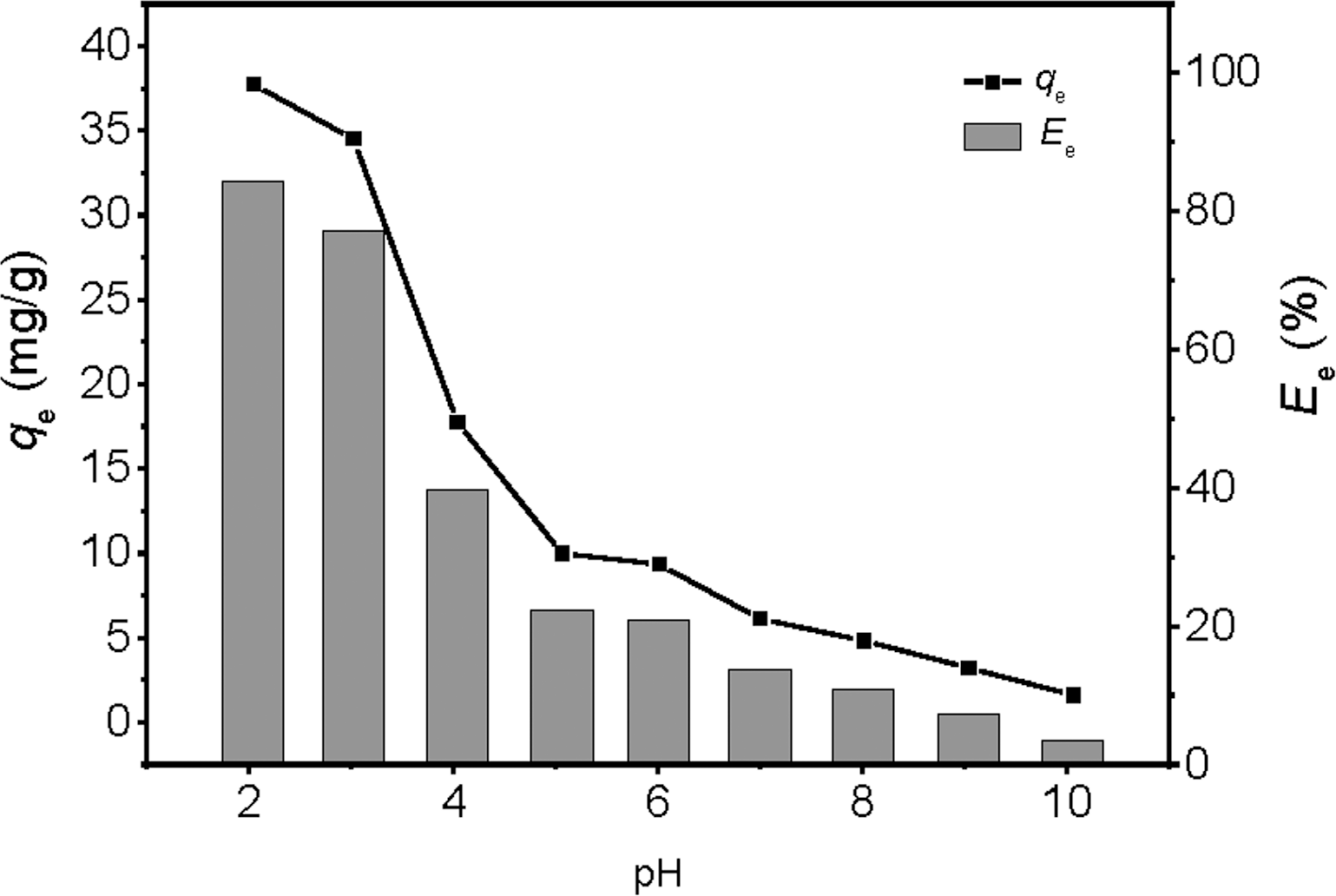
Effect of solution pH on Cr(VI) adsorption onto the EDA-GO@Fe_3_O_4_: (initial Cr(VI) concentration = 10 mg/L; sorbent dose = 2 mL; temperature = 25°C; time = 8 h).

Solution pH is a very important factor for affecting sorption efficiency. In Fig 3, the sorption capacities and adsorption percentages of EDA-GO@Fe_3_O_4_ decreased significantly when the solution pH value increased from 2 to 10. For instance, the adsorption capacity and adsorption percentages were 37.73 mg/g and 84% at pH = 2, but only 1.60 mg/g and 4% at pH = 10, respectively. This result indicated clearly that the adsorption process was pH dependent, which may be due to that pH can affect the surface binding sites of EDA-GO@Fe_3_O_4_ and the aqueous chemistry. HCrO_4_^−^, KCrO_4_^−^, H_2_CrO_4(aq)_, CrO_4_^2−^, and Cr_2_O_7_^−^ are the main Cr(VI) species at low pH levels, and the HCrO_4_^−^ is the predominant form [1, 31]. The zeta potentials of EDA-GO@Fe_3_O_4_ were determined at different pH (ZEN3690, Malvern, UK), and the results is shown in S1 Fig. The pH_pzc_ value was 4.73. At pH < 4.73, EDA-GO@Fe_3_O_4_ surfaces were positively charged due to protonation reactions on the functional groups of the composite. Therefore, the positively charged EDA-GO@Fe_3_O_4_ was more likely to attract negatively charged Cr(VI) ions (HCrO_4_^−^) electrostatically. However, At pH > 4.73, the negative charge on the EDA-GO@Fe_3_O_4_ surface increased because of the deprotonation of the groups at high OH^−^ concentrations [32]. Therefore, the electrostatic repulsion between EDA-GO@Fe_3_O_4_ and Cr(VI) ions (CrO_4_^2−^) was very strong, reducing the Cr(VI) adsorption capacity. In addition, at higher pH levels, as OH^−^ concentrations increased the OH^−^ could compete with CrO_4_^2−^ ions for adsorption sites, decreasing the Cr (VI) adsorption capacity [33].

### Effect of adsorbent dose on adsorption performance

S2 Fig illustrates the influence of adsorbent dose on the adsorption process of the EDA-GO@Fe_3_O_4_ for Cr(VI) ions. Adsorption capacity decreased as the EDA-GO@Fe_3_O_4_ dose increased from 1 mL to 8 mL. This may have been due to the fact that the higher EDA-GO@Fe_3_O_4_ dose provided more adsorption sites for Cr(VI) ions, but the amount of Cr(VI) ions in the system was constant, therefore the Cr(VI) ions were not sufficient for all adsorption sites, resulting in the decrease of adsorption capacity. Increasing EDA-GO@Fe_3_O_4_ dosage might also result in the aggregation of adsorbent particles, thereby decreasing the total surface area of the EDA-GO@Fe_3_O_4_ composite and increasing the diffusion path length of the Cr(VI) ions [34].

### Effect of foreign anions

S3 Fig shows the effect of 0.01 M Cl^−^, NO_3_^−^, ClO_4_^−^, and SO_4_^2−^ on Cr(VI) adsorption by EDA-GO@Fe_3_O_4_ at pH 2. We can see that these foreign anions have different effects on the Cr(VI) removal. The adsorption capacity of EDA-GO@Fe_3_O_4_ for Cr(VI) ions is the highest in the system with addition of ClO_4_^−^, which may be due to that the ClO_4_^−^ ions might not interact with the adsorption sites on the EDA-GO@Fe_3_O_4_ surfaces. The Cr(VI) adsorption capacity of EDA-GO@Fe_3_O_4_ in the present of 0.01 M NO_3_^−^ was lower than those in the systems with Cl^−^ and SO_4_^2−^, which may be mainly ascribed to the NO_3_^−^ competing with the HCrO_4_^−^ ions for the adsorption sites [31]. Besides, the NO_3_^−^ might decrease the zeta potentials of the EDA-GO@Fe_3_O_4_, which reduced the electrostatic attraction forces between the negative HCrO_4_^−^ and the positively charged surfaces of EDA-GO@Fe_3_O_4_, thereby decreasing the Cr(VI) removal[35, 36].

### Desorption and regeneration analysis

In order to determine the reusability of EDA-GO@Fe_3_O_4_, the adsorption-desorption cycles were conducted for four times, and the results are illustrated in S4 Fig. After adsorption experiments, the EDA-GO@Fe_3_O_4_ was regenerated by 0.1 M NaOH, and then rinsed with Milli-Q water. From S4 Fig, the adsorption capacity of EDA-GO@Fe_3_O_4_ for Cr(VI) decreased slightly from 39.67 mg/g to 34.83 mg/g after four cycling runs, which indicated that EDA-GO@Fe_3_O_4_ shows good stability in the adsorption process.

### Adsorption kinetics

**Fig 4:**
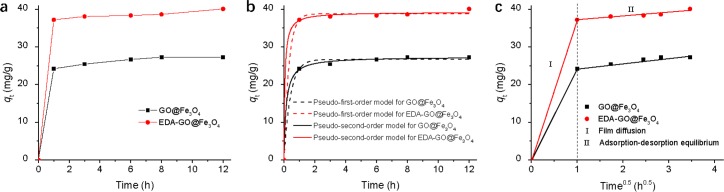
(a) Time profiles of Cr(VI) adsorption with GO@Fe_3_O_4_ and EDA-GO@Fe_3_O_4_; Kinetics of Cr(VI) adsorption by fitting (b) pseudo-first-order and pseudo-second-order models, and (c) intraparticle diffusion model, respectively (initial Cr(VI) concentration = 10 mg/L; sorbent dose = 2 mL; temperature = 25°C; pH = 2).

Fig 4 demonstrates the adsorption kinetics of Cr(VI) ions by GO@Fe_3_O_4_ and EDA-GO@Fe_3_O_4_. Fig 4A shows that adsorption equilibrium could be reached at 1 h for GO@Fe_3_O_4_ and EDA-GO@Fe_3_O_4_. The adsorption of Cr(VI) ions did not increase significantly after 1 h, which might have been due to the complete occupation of available adsorption sites on the surfaces of GO@Fe_3_O_4_ and EDA-GO@Fe_3_O_4_ [37]. Fig 4A also shows that the adsorption capacities of EDA-GO@Fe_3_O_4_ for Cr(VI) ions were higher than those of GO@Fe_3_O_4_, indicating that the ethylenediamine grafted on the GO@Fe_3_O_4_ surface enhances the adsorption capacity of EDA-GO@Fe_3_O_4_.

The adsorption experimental data was interpreted by the kinetics models of pseudo-first-order, pseudo-second-order, and intra-particle diffusion (Fig 4B and 4C). The parameters of pseudo-first-order and pseudo-second-order adsorption kinetics are summarized in Table 1. In both Fig 4B and Table 1, the correlation coefficient (*R*^2^) values of the GO@Fe_3_O_4_ and EDA-GO@Fe_3_O_4_ for the pseudo-second-order model were higher than those for the pseudo-first-order model. In addition, the calculated *q*_e_ values for the pseudo-second-order model were very close to the experimental data. These results indicate that the pseudo-second-order model is more suitable to describe the adsorption experimental data, indicating that chemical adsorption reaction is the dominant rate-limiting step for both adsorption processes.

**Table 1:** Adsorption kinetics parameters for Cr(VI) adsorption onto GO@Fe_3_O_4_ and EDA-GO@Fe_3_O_4_.

Adsorbents	Pseudo-first-order			Pseudo-second-order		
	*q*_e,1_	*k*_1_	*R*^2^	*q*_e,2_	*k*_2_	*R*^2^
GO@Fe_3_O_4_	26.675	2.367	0.995	27.382	0.258	0.998
EDA-GO@Fe_3_O_4_	38.791	3.198	0.997	39.247	0.424	0.998

Fig 4C illustrates the *q*_t_ vs. *t*^0.5^ plot. The multi-linear plot can be separated into two largely linear regions, indicating that intra-particle diffusion of the Cr(VI) ions was not the rate-controlling step for the overall adsorption process. The first region in Fig 4C (labeled “I”) might be assigned to film diffusion corresponding to transportation of Cr(VI) ions from the aqueous solution to the external surfaces of GO@Fe_3_O_4_ and EDA-GO@Fe_3_O_4_. The second region (labeled “II”) includes the gradual sorption and equilibrium stages [38].

### Adsorption isotherm

**Fig 5:**
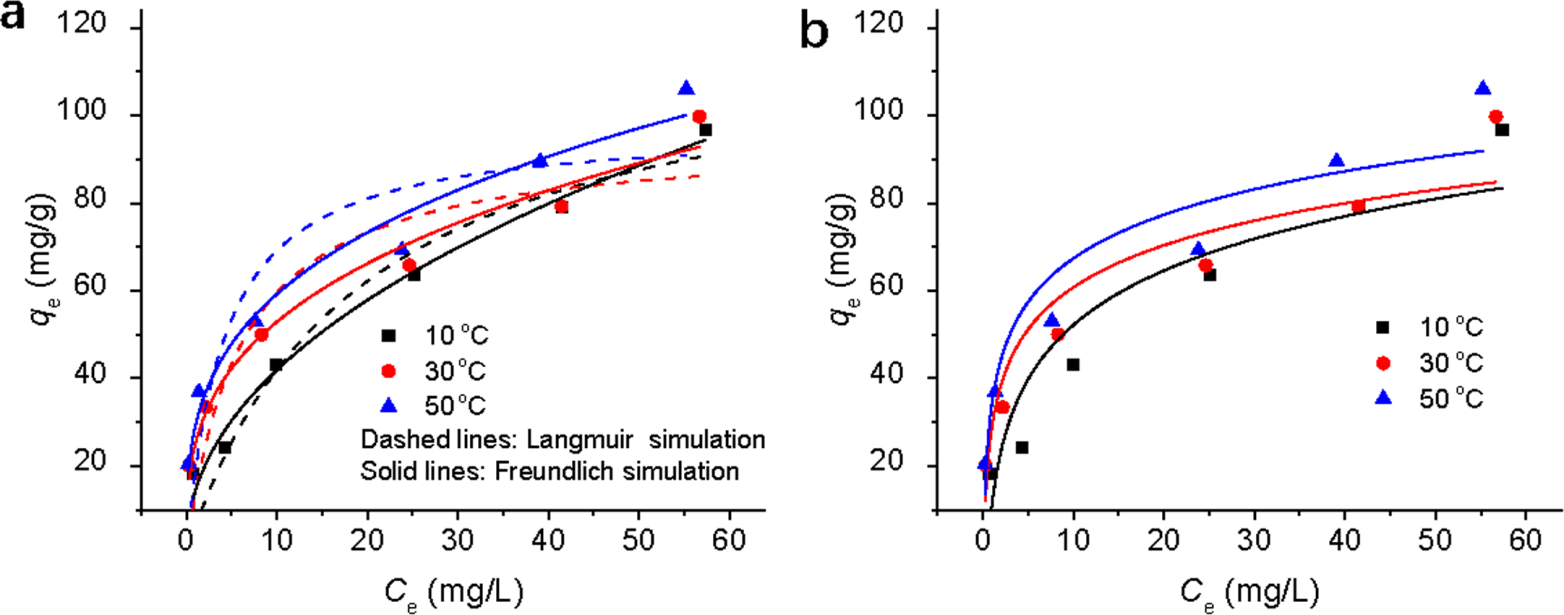
(a) Langmuir and Freundlich and (b) Temkin plots for Cr(VI) ions onto EDA-GO@Fe_3_O_4_ at 10, 30, and 50°C, respectively (time = 8 h; sorbent dose = 223 mg/L; pH = 2).

The decontamination of Cr(VI) ions by EDA-GO@Fe_3_O_4_ was studied at 15°C, 30°C, and 50°C to determine the relative parameters of the adsorption isotherms, and the results are demonstrated in Fig 5. The adsorption capacities of EDA-GO@Fe_3_O_4_ for Cr(VI) ions increased when the temperature increased from 10 to 50°C, implying an endothermic process. The nonlinear form of Langmuir, Freundlich, and Temkin adsorption isotherms at different temperatures are also illustrated in Fig 5. The parameters for the three isotherm models are demonstrated in Table 2. The Freundlich model clearly describes the isotherm adsorption data better than the Langmuir and Temkin models within the studied temperature range. The Freundlich constants of *n* (2.150 for 10°C, 3.086 for 30°C, and 3.259 for 50°C) are within the beneficial adsorption range (1–10) [39], indicating that EDA-GO@Fe_3_O_4_ can be applied as an effective adsorbent.

**Table 2:** Isotherm parameters for Cr(VI) ions adsorption onto EDA-GO@Fe_3_O_4_.

Models	Parameters	Temperature (°C)		
		10	30	50
Langmuir	*q*_max_	120.327	95.222	97.808
	*K*_L_	0.054	0.167	0.241
	*R*^2^	0.931	0.813	0.781
Freundlich	*n*	2.150	3.086	3.259
	*K*_F_	14.363	25.085	29.222
	*R*^2^	0.984	0.970	0.970
Temkin	*a*_T_	1.848	8.083	10.951
	*b*_T_	0.132	0.182	0.187
	*R*^2^	0.844	0.869	0.885

### FFD for assessing the effects of multiple factors on adsorption

The profiles of Cr(VI) removal by EDA-GO@Fe_3_O_4_ under varying levels of multiple experimental factors are illustrated in S5 Fig. Higher Cr(VI) removal was clearly found in Runs 3 and 11. Both of these experiments had low pH values (2) and adsorbent dose (1 mL) and high initial concentration of Cr(VI) ions (80 mg/L). In contrast, runs 1, 7, 15, and 16, which involved high pH values (10) and low Cr(VI) concentration (20 mg/L), showed lower Cr(VI) removal efficiency.

**Fig 6:**
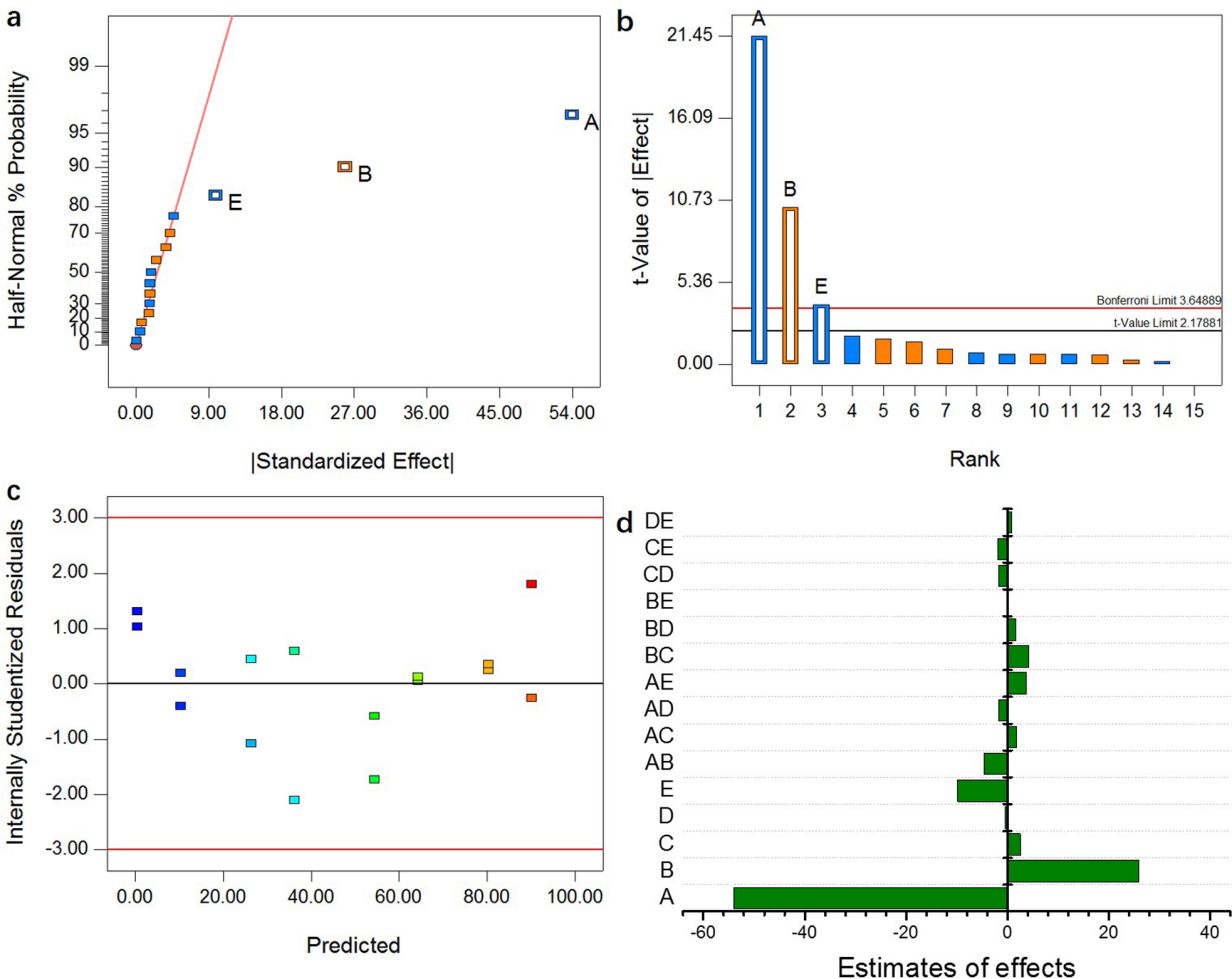
(a) Half-normal probability plot; (b) Pareto chart; (c) Plots of internally standardized residuals with predicted values; (d) Identification of main effective factors and interaction factors on Cr(VI) adsorption by EDA-GO@Fe_3_O_4_: (A) pH; (B) Cr(VI) concentration; (C) Temperature; (D) Time; (E) Adsorbent dose.

The effects of experimental factor variation and factor interactions on Cr(VI) decontamination were evaluated by significance testing of the FFD model [40]. The half-normal probability plot is shown in Fig 6A. Significant effects were observed in connection with variations of factors A, B, and E, while factor interactions have nonsignificant effects on the adsorption process. The Pareto chart (Fig 6B) was used to verify the results. The effect terms of A, B, and E are above the Bonferroni limit, indicating that these factorial effects are important factors in the removal process. Based on these findings, and the calculated coefficients, the model for predicting Cr(VI) removal can be represented by the following equation in terms of the factors:

$$q_{e}=74.13522—6.75045\times \mathrm{pH}+0.43143\times B—4.92608\times E$$ (9)

The plot of normal probability of residuals for Cr(VI) decontamination is demonstrated in S6 Fig. All internally studentized residuals lie close to a straight line, indicating a normal pattern for the regression residuals [41]. S7 Fig shows predicted values versus actual values. The predicted values are very close to the experimental measurements, implying that the Cr(VI) adsorption process can be predicted by the FFD models obtained. Fig 6C shows that the internally studentized residuals were equally scattered between −3 and +3, which indicated that the obtained FFD model in this study was adequate [40].

The identification of important factors, and factor interaction on Cr(VI) adsorption by EDA-GO@Fe_3_O_4_, is illustrated in Fig 6D. The factors with negative effects were A (−54.00), D (−0.51), and E (−9.85), while positive estimates of the effects of B (25.89) and C (2.52) were observed. This indicates that factor A is very important in the removal process. The effects of the six selected factors on Cr(VI) adsorption by EDA-GO@Fe_3_O_4_ were found to lie in the order A > B > E > C > D.

**Fig 7:**
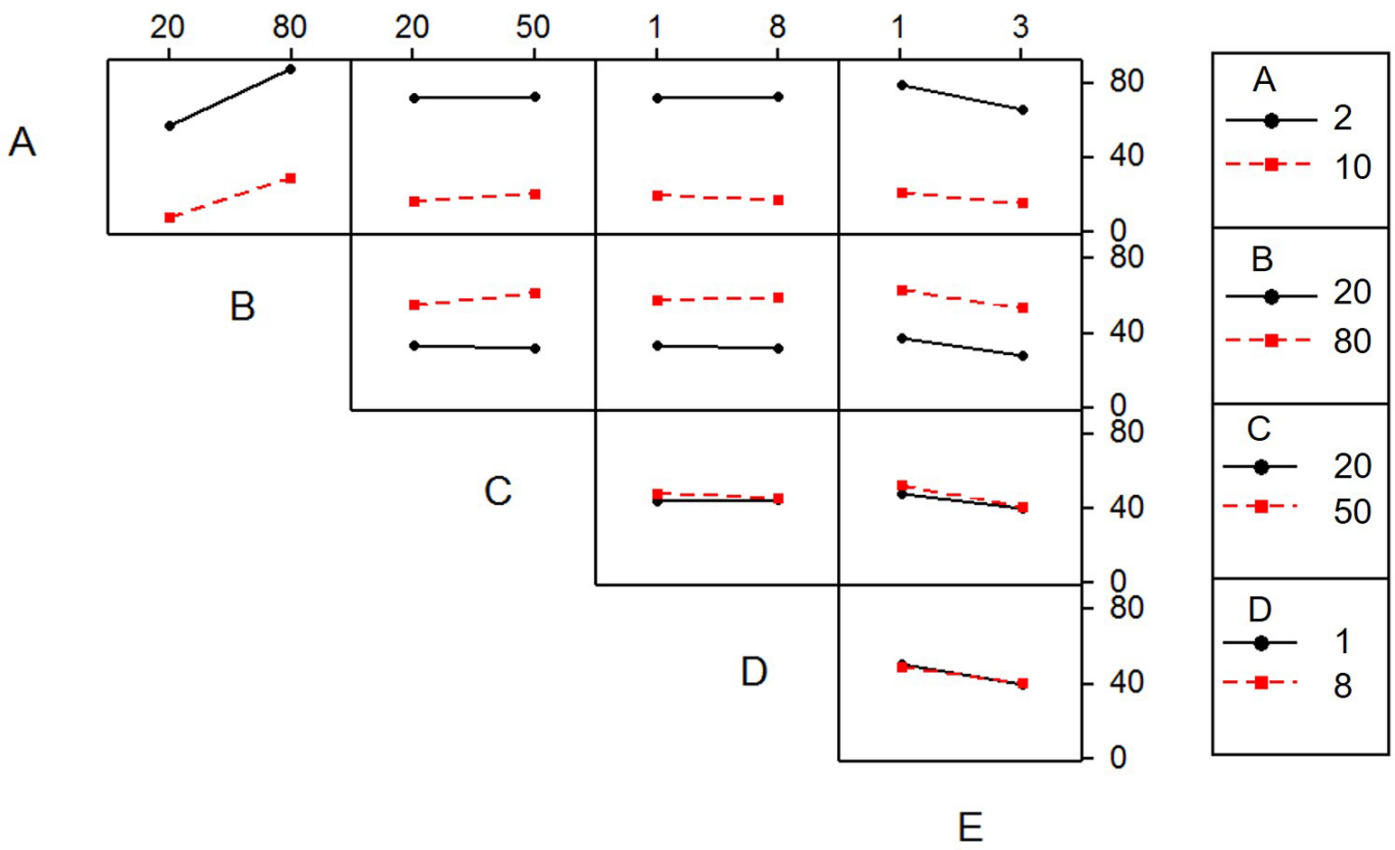
Interaction effects plot for Cr(VI) decontamination: (A) pH; (B) Cr(VI) concentration; (C) Temperature; (D) Time; (E) Adsorbent dose.

Fig 7 shows factor interaction effects for Cr(VI) decontamination. Lines in cells AB, AE, and BC were non-parallel, indicating that these factors could affect each other significantly [35]. In row A, Cr(VI) adsorption decreased slightly with the increase of pH values, indicating that factor A had a large impact on Cr(VI) decontamination. The two lines in row D coincided, suggesting that factor D had a slight or no impact on Cr(VI) removal.

## Conclusions

The HRTEM, EDS, FT-IR, TG-DSC, and XPS analyses indicate successful preparation of a novel ethylenediamine graft to a magnetic graphene oxide composite (EDA-GO@Fe_3_O_4_) using a simple chemical synthesis method. EDA-GO@Fe_3_O_4_ showed higher adsorption capacity for Cr(VI) ions than unmodified GO@Fe_3_O_4_, and the removal process was found to be affected by the operational parameters. The sorption capacities of EDA-GO@Fe_3_O_4_ decreased significantly with increased pH values due to the fact that pH affected both the aqueous chemistry and the surface binding-sites of EDA-GO@Fe_3_O_4_. The foreign anion of NO_3_^−^ can compete with the HCrO_4_^−^ ions for the adsorption sites. The EDA-GO@Fe_3_O_4_ has a good reusability. Adsorption equilibrium was reached within 1 h for both GO@Fe_3_O_4_ and EDA-GO@Fe_3_O_4._ The pseudo-second-order model is suitable for being used to describe the adsorption kinetics experimental data. The Freundlich model fits the adsorption isotherm data better than the Langmuir and Temkin models. Operational parameters A (pH), B (Cr(VI) concentration), and E (adsorbent dose) have significant effects on the removal process. The effects of the six selected factors on the decontamination process follow the order of A > B > E > C > D. The combined factors AB, AE, and BC have larger effects on Cr(VI) ions removal than other interactions. Consequently, the experimental results indicate that EDA-GO@Fe_3_O_4_ will have broad applications in cleaning up chromium pollution.

## Supporting information

S1 Table Experimental design matrix of the 2^5−1^ FFD with resolution V for Cr(VI) adsorption ontoEDA-GO@Fe_3_O_4_. (DOCX)

S1 Fig The zeta potentials of EDA-GO@Fe_3_O_4_ at different pH. (DOCX)

S2 Fig Decontamination of Cr(VI) with EDA-GO@Fe_3_O_4_ as a function of sorbent dosage (initial Cr(VI) concentration = 10 mg/L; time = 8 h; temperature = 25°C; pH = 2). (DOCX)

S3 Fig Effect of the 0.01 M foreign anions (initial Cr(VI) concentration = 10 mg/L; time = 8 h; temperature = 25°C; pH = 2). (DOCX)

S4 Fig Four desorption/adsorption cycles of EDA-GO@Fe_3_O_4_ for Cr(VI) removal. (DOCX)

S5 Fig Experimental data obtained from the FFD experiments. (DOCX)

S6 Fig Plot of normal probability of residuals for Cr(VI) adsorption onto EDA-GO@Fe_3_O_4_. (DOCX)

S7 Fig Comparison of predicted and experimental adsorption capacities of Cr(VI) by EDA-GO@Fe_3_O_4_. (DOCX)
