# Supplementary material for: Ethylenediamine grafted to graphene oxide@Fe3O4 for chromium(VI) decontamination: Performance, modelling, and fractional factorial design
Source: PLoS One. 2017 Oct 30;12(10):e0187166. doi: 10.1371/journal.pone.0187166 (PMC5662183; doi:10.1371/journal.pone.0187166)
Supplement: S1 Table — (DOCX) [file pone.0187166.s001.docx]

**S1 Table Experimental design matrix of the 2^5−1^ FFD with resolution V for Cr(VI) adsorption onto EDA-GO@Fe_3_O_4_**

| Run number | Values of independent variables* | | | | |
| --- | --- | --- | --- | --- | --- |
|  | A | B | C | D | E |
| 1 | 10 | 20 | 50 | 8 | 1 |
| 2 | 2 | 20 | 50 | 8 | 3 |
| 3 | 2 | 80 | 20 | 1 | 1 |
| 4 | 2 | 80 | 20 | 8 | 3 |
| 5 | 10 | 80 | 50 | 1 | 1 |
| 6 | 10 | 80 | 20 | 1 | 3 |
| 7 | 10 | 20 | 20 | 1 | 1 |
| 8 | 10 | 80 | 20 | 8 | 1 |
| 9 | 2 | 20 | 50 | 1 | 1 |
| 10 | 2 | 20 | 20 | 1 | 3 |
| 11 | 2 | 80 | 50 | 8 | 1 |
| 12 | 10 | 80 | 50 | 8 | 3 |
| 13 | 2 | 80 | 50 | 1 | 3 |
| 14 | 2 | 20 | 20 | 8 | 1 |
| 15 | 10 | 20 | 50 | 1 | 3 |
| 16 | 10 | 20 | 20 | 8 | 3 |

* A: pH; B: Cr(VI) concentration (mg/L); C: Temperature (°C); D: Time (h); E: Adsorbent dose (mL)
