# Supplementary material for: Ethylenediamine grafted to graphene oxide@Fe3O4 for chromium(VI) decontamination: Performance, modelling, and fractional factorial design
Source: PLoS One. 2017 Oct 30;12(10):e0187166. doi: 10.1371/journal.pone.0187166 (PMC5662183; doi:10.1371/journal.pone.0187166)
Supplement: S3 Fig — (DOCX) [file pone.0187166.s004.docx]

**S3 Fig.** **Effect of the 0.01 M foreign anions (initial Cr(VI) concentration = 10 mg/L; time = 8 h; temperature = 25 °C; pH = 2)**
