# Supplementary material for: Ethylenediamine grafted to graphene oxide@Fe3O4 for chromium(VI) decontamination: Performance, modelling, and fractional factorial design
Source: PLoS One. 2017 Oct 30;12(10):e0187166. doi: 10.1371/journal.pone.0187166 (PMC5662183; doi:10.1371/journal.pone.0187166)
Supplement: S4 Fig — (DOCX) [file pone.0187166.s005.docx]

**S4 Fig.** **Four desorption/adsorption cycles of EDA-GO@Fe_3_O_4_ for Cr(VI) removal**
