# Supplementary figures and images for: Ethylenediamine grafted to graphene oxide@Fe3O4 for chromium(VI) decontamination: Performance, modelling, and fractional factorial design
Source: PLoS One. 2017 Oct 30;12(10):e0187166. doi: 10.1371/journal.pone.0187166 (PMC5662183; doi:10.1371/journal.pone.0187166)

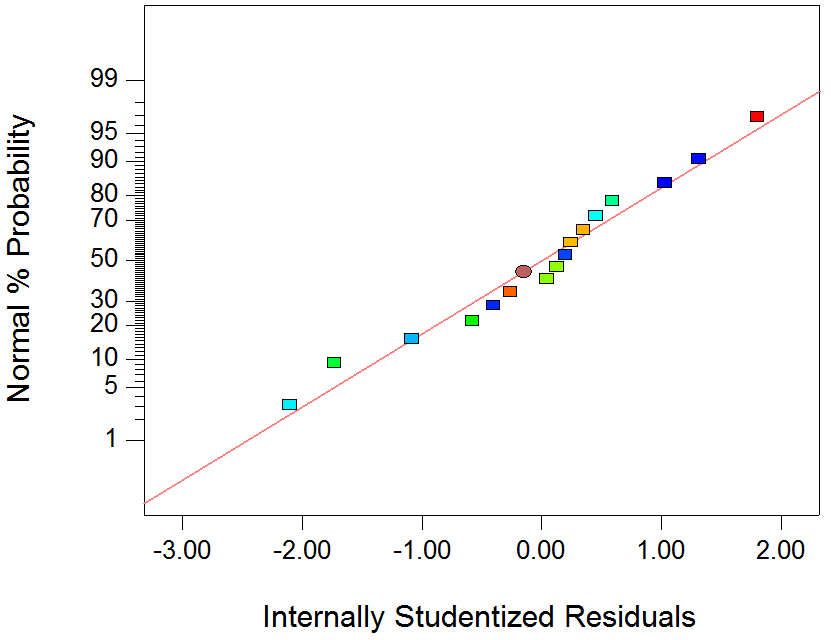


**S6 Fig. Plot of normal probability of residuals for Cr(VI) adsorption onto EDA-GO@Fe3O4.**

Supplement: S6 Fig — (DOCX) [file pone.0187166.s007.docx]

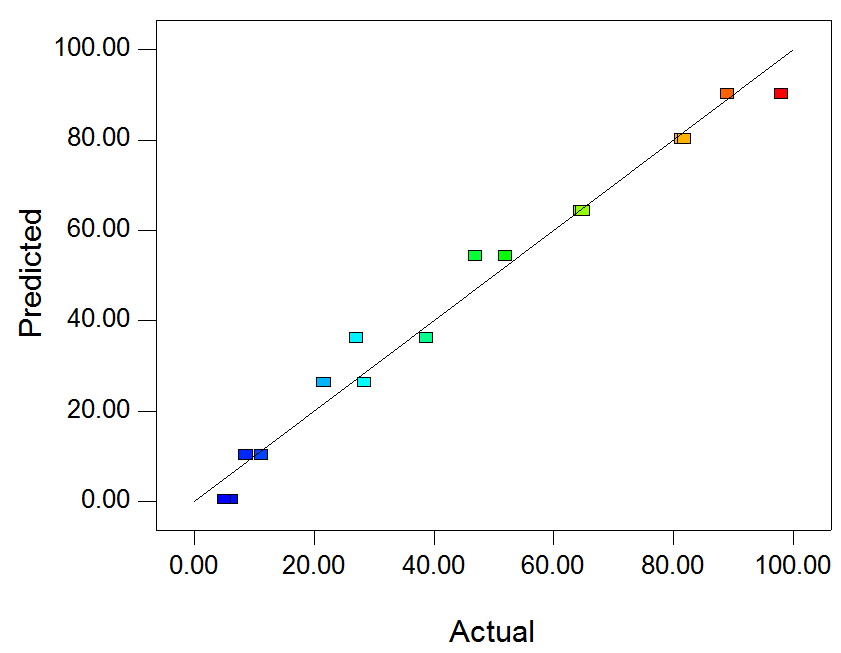


**S7 Fig. Comparison of predicted and experimental adsorption capacities of Cr(VI) by** **EDA-GO@Fe3O4.**

Supplement: S7 Fig — (DOCX) [file pone.0187166.s008.docx]
